# Report of the Post-Mortem Examination of the Body of Charles J. Guiteau

**Published:** 1882-07

**Authors:** D. S. Lamb

**Affiliations:** Army Medical Museum, Washington, D. C.


REPORT OF THE POST-MORTEM EXAMINATION OF THE BODY OF CHARLES J. GUITEAU, (who Died by Hanging June 30, 1882, at the United States Jail, Washington, D. C., in Execution of Judicial Sentence.)

The following physicians were present:

Dr. Noble Young, Physician to the Jail; Dr. A. McWilliam, his assistant; Dr. C. H. Nichols, Supt. Insane Asylum at Bloomingdale, N. Y.; Dr. A. E. McDonald, Supt. Insane Asylum, New York City; Dr. W. W. Godding, Supt. Government Insane Asylum, District of Columbia, and Drs. Witmer and Patterson, his assistants; Dr. Geo. L. Porter, Bridgeport, Ct.; Dr. Johnston Eliot, Washington, D. C.; Dr. D. R. Hagner, Washington, D. C.; Dr. Robert Reyburn, Washington, D. C.; Dr. W. J. Morton, Editor of *Journal of Nervous and Mental Diseases*, New York City; Dr. C. L. Dana, New York City; Dr. C. K. Mills, Philadelphia, Pa.; Dr. D. C. Patterson, Coroner, Washington, D. C.; Dr. J. F. Hartigan, Physician to Coroner; Dr. C. H. A. Kleinschmidt, Washington, D. C.; Dr. P. J. Murphy, Washington, D. C.; Dr. Z. T. Sowers, Washington, D. C.; Dr. F. B. Loring, Eye and Ear Dispensary, Washington, D. C.; Dr. D. S. Lamb, Acting Ass't Surg., U. S. A., and Dr. J. C. McConnell, Microscopist, and Mr. Ernst Schafhirt, Anatomist, Army Medical Museum.

The custodian of the body, Rev. Dr. W. W. Hicks, having requested me to make the examination, and this request being in accordance with one previously made by Dr. Young, I had prepared the following course of procedure, with the object of securing the most exact and satisfactory results: The external appearances of the body should first be noted; the cavities, except the spinal, should be opened and their contents examined; that after the removal of the brain, and its examination without incision, it should be transferred, properly guarded and protected, to the Army Medical Museum, where, through the courtesy of the Curator, Surgeon D. L. Huntington, U. S. Army, it would be photographed, and then a cast be taken; that its internal structure should then be observed, and portions set apart for microscopical examination; and that the entire operation should be completed, as far as possible, the same day, to enable physicians resident elsewhere, present by invitation, to return promptly to their homes; and that the notes should be taken in duplicate.

Shortly previous to the examination, it was agreed between Rev. Dr. Hicks and the United States District Attorney, Geo. B. Corkhill, that the charge of the removal, preservation, and disposition of the brain should be vested jointly in Drs. Lamb, Sowers and Hartigan, and that the microscopical examination should be made by parties to be selected by the gentlemen making the agreement.

The examination was then conducted by me, assisted by Drs. Hartigan and Sowers and Mr. Schafhirt, and as near as possible in the order proposed. Drs. Kleinschmidt, Patterson, and others, took notes, and Mr. Charles Trought, of the Museum, the photographs.

By reason of delays, for which neither I nor my assistants were responsible, the examination was not begun until 2:30 P. M. (one hour and a half after death), in consequence of which the photographing was less successful, and a cast was impracticable.

The body, which was of a faint yellowish tint, was that of a man about five feet seven inches in height, and weighed one hundred and forty-five pounds.

The eyes were examined by Dr. Loring, who reported that the pupils were slightly and equally dilated; the vitreous was cloudy, and the fundus indistinguishable. The conjunctiva of the left eye was congested. He repeated the examination two hours later, and noticed an appearance as of transverse fracture of the lenses.

A small white scar, directed obliquely downward, forward and to the left, and confined to the scalp, was observed midway between the top of the left ear and median line of the head.

There was a yellowish furrow, a few lines in width, extending around the neck in a direction downward and forward, in line of rope. On dissection, the sterno-cleido-mastoid muscles were found to be torn in two, about half way between their points of origin and insertion. The thyro-hyoid ligament was also ruptured, and the hyoid bone and thyroid cartilage widely separated. The large blood-vessels were not injured; neither was there fracture nor dislocation of the vertebræ.

The sexual organs were well developed; the mouth of the urethra was moist.* There was also phimosis, on reducing which, a considerable accumulation of smegma was observed.

*Skull.*—The right parietal bone was slightly flattened over a space about two inches square, just back of the fronto-parietal suture and to the right of the interparietal; there was a slight flattened elevation on the corresponding internal surface of the calvaria. The frontal suture was obliterated, the others quite distinct. A number of Pacchionian depressions were observed near the groove for the longitudinal sinus. In thickness the skull presented nothing remarkable.

*Membranes of the Brain.*—The dura mater was firmly adherent to the anterior portion of the calvaria in the vicinity of the longitudinal sinus.

There were adhesions of the dura also to the base of the skull; they were quite firm, and situated in the several fossæ, and most marked in the deeper parts of the fossæ, where also there were small patches, abruptly limited, of immovable arborescent congestion, with, however, no attendant thickening or pigmentation; this *stagnation* was again most marked in the left anterior and middle fossæ. There was no congestion of the dura except at the points just noted.

The dura and pia mater were adherent to each other and to the brain on both sides along a limited portion of the longitudinal fissure, in the vicinity of Pacchionian granulations.

The dura was slightly thickened along the longitudinal sinus. It was also slightly thickened and opaque along a portion of the line of the middle meningeal artery on each side.

The arachnoid of the upper convexity of the brain presented in many places, where it covered the sulci, small patches of thickening and opacity; elsewhere it was normal.

The pia mater was anæmic anteriorly; posteriorly there was slight hypostasis.

The cerebral vessels appeared to be normal in all respects.

---

* During the suspension of the body, erection of the penis took place, and the clothing was afterward found to be moist.

The orbital plates were well arched, and presented many conical eminences of large size. There was no roughening anywhere of the inner surface of the skull.

*The brain* was firm. Its weight, including the cerebrum, cerebellum, pons and medulla, and a portion of the dura, was 49½ ounces. It was slightly flattened in the region corresponding to the flattening of the parietal bone above mentioned. On section of the cerebrum there was an appearance as of slight thinning of the gray cortex; the measurements taken, however, gave depths of one-sixteenth to one-eighth inch in close proximity to each other. The white substance was almost absolutely anæmic. The cerebellum and island of Reil were both *covered* on each side.

*The fissures.*—The fissures generally presented a considerable depth; in many places, as in the right fissure of Rolando, amounting to seven-eighths inch.

The right fissure of Sylvius was typical; the left was separated from the first temporal fissure by a slight bridge deeply situated. The right fissure of Rolando did not connect with the fissure of Sylvius; the left was separated only by a small bridge deeply situated. Both were separated from the longitudinal fissure.

The first frontal fissure on the right side was not connected with that of Rolando, but at the posterior part was crossed by a secondary fissure. The same on the left side, except that the fissure was crossed by a small bridge near its center. The second and third frontal fissures presented nothing remarkable. There were numerous secondary fissures.

The præcentral and retrocentral fissures, on each side, were well defined, and were unconnected with other fissures. The interparietal fissure, on each side, terminated in the transverse occipital, separated only by a slight bridge. The parieto-occipital was well marked on each side. The transverse occipital fissure on the right side was ill defined; it began on the median surface and extended well outward. The first temporal fissure was well developed on the right side; on the left, was not of the usual length. Wernicke's fissure was well marked on the left side, but not confluent.

The calloso-marginal fissure was double on each side; the upper of the two being probably the true one. On the right, the upper one extended back to the anterior margin of the paracentral lobule; on the left, not quite so far. The lower one extended on the right side to a line about half an inch in front of the parieto-occipital fissure, from which it was separated by a small bridge; on the left side, also, by a bridge of larger size.

*Orbital Surface.*—On the right side were seven fissures, radiating from a circluar fissure surrounding a small isolated convolution; on the left side were five fissures, radiating from a small shallow depression. The left collateral fissure was well defined, extending to the anterior extremity of the temporal lobe; the right was also well marked, but did not extend so far back as the other, and there was an attempt at confluence anteriorly with the temporo-occipital, a small bridge intervening. The left temporo-occipital fissure was well defined.

*The Convolutions.*—The following alone call for remark: The ascending frontal was well defined on each side.

The ascending parietal on the right side was well developed in its lower three-fourths, but narrowed in the upper fourth. On the left side the narrowing was less marked. The island of Reil presented on the right side five fissures and six straight gyri; on the left side seven fissures and eight straight gyri. The paracentral lobe was well marked on the right side; small on the left.

*Thorax and Abdomen.*—The usual median incision was made, and the abdomen opened. There was an extravasation of blood into the right pectoralis major muscle near the second rib. The adipose layer of the abdominal section was one inch in thickness. The dome of the diaphragm extended up to the fourth rib on each side.

There were old pleuritic adhesions at the apex of the right lung; the upper and middle lobes were congenitally united by connective tissue. The lung was normal throughout. There were also old pleuritic adhesions of the left lung to the diaphragm and between its lobes; three small tubercle-like, pigmented patches were observed in the upper lobe. The heart weighed ten and three-quarter ounces; its muscular substance was apparently normal. There was an abundance of fat upon its anterior surface, and a villous patch of old pericarditis near the apex of the left ventricle. The right ventricle contained a little blood just forming a clot.* The valves were normal. The aorta was slightly atheromatous for a short distance above the valves.

All of the abdominal viscera presented large accumulations of fat. They were normally situated. The liver was congested; the gall bladder contained a little bile; the spleen was lobulated and enlarged; it weighed eighteen ounces; the capsule was bluish, the substance brown; the Malpighian bodies hypertrophied;† the pancreas was normal; the stomach contained food;‡ the intestines appeared normal; were not opened; the kidneys were congested; there was a small superficial serous cyst on the right one. The bladder contained a considerable quantity of urine.

The results of the microscopic examinations will be reported hereafter. D. S. LAMB.

*Army Medical Museum, Washington, D. C., July* 4, 1882.

* A considerable quantity of dark blood ran out of the heart in the separation of the heart and lungs.

† Dr. Young states that the man was subject to malarial attacks while in the jail.

‡ He had eaten a dinner about an hour and a half before the execution.

